# Inhibition of the Water Oxidizing Complex of Photosystem II and the Reoxidation of the Quinone Acceptor Q_A_^−^ by Pb^2+^

**DOI:** 10.1371/journal.pone.0068142

**Published:** 2013-07-04

**Authors:** Ahmed Belatik, Surat Hotchandani, Robert Carpentier

**Affiliations:** Groupe de Recherche en Biologie Végétale, Université du Québec à Trois-Rivières, Trois-Rivières, Québec, Canada; University of Hyderabad, India

## Abstract

The action of the environmental toxic Pb^2+^ on photosynthetic electron transport was studied in thylakoid membranes isolated from spinach leaves. Fluorescence and thermoluminescence techniques were performed in order to determine the mode of Pb^2+^ action in photosystem II (PSII). The invariance of fluorescence characteristics of chlorophyll a (Chl a) and magnesium tetraphenylporphyrin (MgTPP), a molecule structurally analogous to Chl a, in the presence of Pb^2+^ confirms that Pb cation does not interact directly with chlorophyll molecules in PSII. The results show that Pb interacts with the water oxidation complex thus perturbing charge recombination between the quinone acceptors of PSII and the S_2_ state of the Mn_4_Ca cluster. Electron transfer between the quinone acceptors Q_A_ and Q_B_ is also greatly retarded in the presence of Pb^2+^. This is proposed to be owing to a transmembrane modification of the acceptor side of the photosystem.

## Introduction

Heavy metals play essential cofactor roles as structural and catalytic components of enzymes in many physiological processes required for the normal development of plants. Over the course of evolution, plants have developed different mechanisms that control and respond to the intake and accumulation of both essential and nonessential heavy metals. However, some heavy metals such as lead can be highly toxic to cells and cell organelle functions even at very low concentrations. Although the influence of excessive dose of heavy metals on the photosynthetic activity of plants has been studied in many cultivated species (1–3), the mechanism of heavy metal toxicity on photosynthesis is still a matter of great debate. Some evidence points to their involvement as inhibitors of electron transport in light reactions (4–5) and in the inhibition of enzyme activity in dark reactions by the direct blocking of protein functions or displacement of endogenous metals (6–7).

Lead, found in the environment, comes from both natural and anthropogenic sources. The metal is present in the soil, but also in all other environmental compartments: water, air and even living beings (8). The toxicity of a metal depends on its chemical state as well as on environmental factors (9–10). In soil, Pb can be found in ionic form, or bound to the soil particles (11). It has two oxidation states, namely 2^+^ and 4^+^. The tetravalent state is a strong oxidant but is not abundant in the environment. The divalent state, on the other hand, is more stable and prominent in the environment (12). The accumulation of Pb from atmospheric deposition or contaminated waste is largely stored in the soil, mainly in the surface layers and, more specifically, in the organic-rich layers (13). However, a small fraction of the metal is also absorbed by living organisms (micro-and meso-organisms, plants …etc.).

The photosystem II (PSII) complex is one of the two membrane-bound large multisubunit chlorophyll–protein complexes (PSII and PSI) of plants, algae and cyanobacteria embedded in the thylakoid membranes. PSII collects light energy, converts it into electro-chemical energy and drives electron transfer from water to PSI. On its acceptor side, PS II electron transport involves two acceptor quinones, Q_A_ and Q_B_ that are arranged around a non-heme iron. This non-heme iron is hexacoordinated by four histidines and two remaining ligand positions are taken by the oxygen atoms of bicarbonate as bidentate ligand (14–15). Further, the study of the effect of bicarbonate has suggested that the non-heme iron plays a role of an electron- transport regulator on the acceptor side of PSII. Although the precise mechanism of this process needs more study, the depletion of bicarbonate results in a decelerating of the electron transfer rate between Q_A_ and Q_B_ (16–20). The water oxidation complex (WOC) is located on the donor side of PSII. It is composed of a Mn_4_Ca cluster where the successive absorption of four quanta by PSII results in the advancement of the S-states cycle from S_0_ → S_1_ → S_2_ → S_3_ → (S_4_) → S_0_. The S_4_-state decays to the S_0_-state after the 4^th^ flash with the concurrent oxygen evolution. The electrons are passed from the WOC to the reaction center P680^+^ through the secondary electron donor, Tyr_Z_ (Tyrosine 161 of D1 subunit) (21).

At present, there are only a few reports regarding the adverse action of Pb^2+^ on the photosynthetic apparatus (22 and references therein). A decline of the photochemical quantum yield of PSII was observed in isolated thylakoid membranes from spinach (23). It was proposed that Pb^2+^ affects oxygen evolution by removing extrinsic polypeptides and/or Ca^2+^ or Cl^−^ ions associated with the water oxidizing complex of PSII (5, 24). Lead cation was also recently shown to affect PSI electron transport presumably due to binding near or at plastocyanin (25).

In this study, we have further investigated the mechanism of the action of Pb^2+^ in thylakoid membranes. The effects of the metal ion on the electron transport were studied using thermoluminescence and fluorescence spectroscopic techniques. Functional assays were used to determine the site of action and consequences of metal ion interaction in the thylakoid membranes and to explore the mode of action of the metal that causes the loss of photosystem II functions.

## Materials and Methods

### Thylakoid Membranes Isolation

Thylakoid membranes were prepared from fresh market spinach leaves (*Spinacia oleracea* L.) as described elsewhere (26), and were stored in the dark in 50 mM Hepes NaOH (pH 7.6), 0.33 M sorbitol, 2 mM EDTA, 1 mM MgCl_2_, 1 mM NaCl, and 10 mM KCl.

### Chlorophyll Fluorescence Induction

Chlorophyll *a* fluorescence induction (FI) measurements were performed at room temperature using the Plant Efficiency Analyser (Hansatech, King’ Lynn, Norfolk, UK). The assay medium consisted of 50 mM Hepes-NaOH (pH 7.6), 0.33 M sorbitol, 2 mM EDTA, 1 mM MgCl_2_, 1 mM MnCl_2_, 10 mM KCl, and 10 mM NaCl with a final Chl concentration of 25 µg mL^−1^ for thylakoid membranes. Red excitation light peaking at 655 nm with an intensity of 1800 µmol m^−2^ s^−1^ was obtained from six light emitting diodes. As the fluorescence signal during the first 40 µs is ascribed to artifacts due to a delay in response time of the instrument, these data were not included in the analysis of FI traces.

### Thermoluminescence

Measurements of thermoluminescence were performed using home-built equipment. The complete description of the design and functional aspects are described elsewhere (27–28). Thylakoid membranes were diluted to a final Chl concentration of 200 µg mL^−1^ in a medium containing 50 mM Hepes-NaOH (pH 7.6), 0.33 M sorbitol, 2 mM EDTA, 1 mM MgCl_2_, 1 mM MnCl_2_, 10 mM KCl, and 10 mM NaCl. About 300 µL of the suspension was added to the sample compartment (15 mm diameter) positioned just above Peltier plate and covered with a Hellma 202-OS disc window. The sample chamber was closed with a holder bearing the light guide connected to the photomultiplier. The sequence of incubation periods and flash illumination was as follows. The samples were pre-incubated for 120 s at 20°C. Then the temperature was brought down to 2°C within 36 s and kept for 60 s**.** Two actinic single turn-over saturating white flashes of about 2 µs pulse width (setting 10, XE-STC, Walz, Germany) were then applied to initiate charge separation in PSII. Finally, a linear warming (0.5°C s^−1^) of the samples in total darkness activated the recombination of PSII charge pairs that can be detected by the appearance of emission bands with characteristic temperature optima (27–28).

### Fluorescence Measurements

Fluorometric experiments were carried out at room temperature 24°C with a Perkin Elmer LS55 Spectrometer equipped with a red-sensitive photomultiplier R928. Samples were excited at 434 nm and fluorescence emission spectra were measured from 600 to 800 nm as described by Rajagopal et al (2003) (29). The excitation and emission spectral widths were fixed at 5 and 2.5 nm, respectively, and emission spectra were corrected according to the photomultiplier sensitivity using the correction factor spectrum provided by Perkin-Elmer.

### Flash-induced Fluorescence Decay Kinetics

In order to examine the reduction and oxidation kinetics of Q_A_, Chl fluorescence rise and its relaxation in the dark were measured with FL3500 Fluorometer (Photon Systems Instruments, Brno, Czech Republic) as described previously (30–31). Thylakoid membranes (Chl concentration of 25 µg ml^−1^) were incubated for 3 min at room temperature in complete darkness without or with 50 µM of DCMU before initiating the fluorescence measurements. Samples were excited with a 20 µs red actinic flash from a LED peaking at 625 nm and prompt fluorescence was measured for 1 min. The first measurement was taken 20 µs after the flash was given. The traces were averaged to estimate the half-life times and amplitudes of the fluorescence decay components using the following three exponential functions:
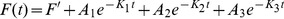
(1)where F(t) is the fluorescence value at time t, k_n_ is the rate constant, A_n_ is the amplitude of the fluorescence relaxation phase, and F′ is the stable minimal fluorescence at the end of the decay.

## Results

### Chlorophyll Fluorescence Induction

**Figure 1:**
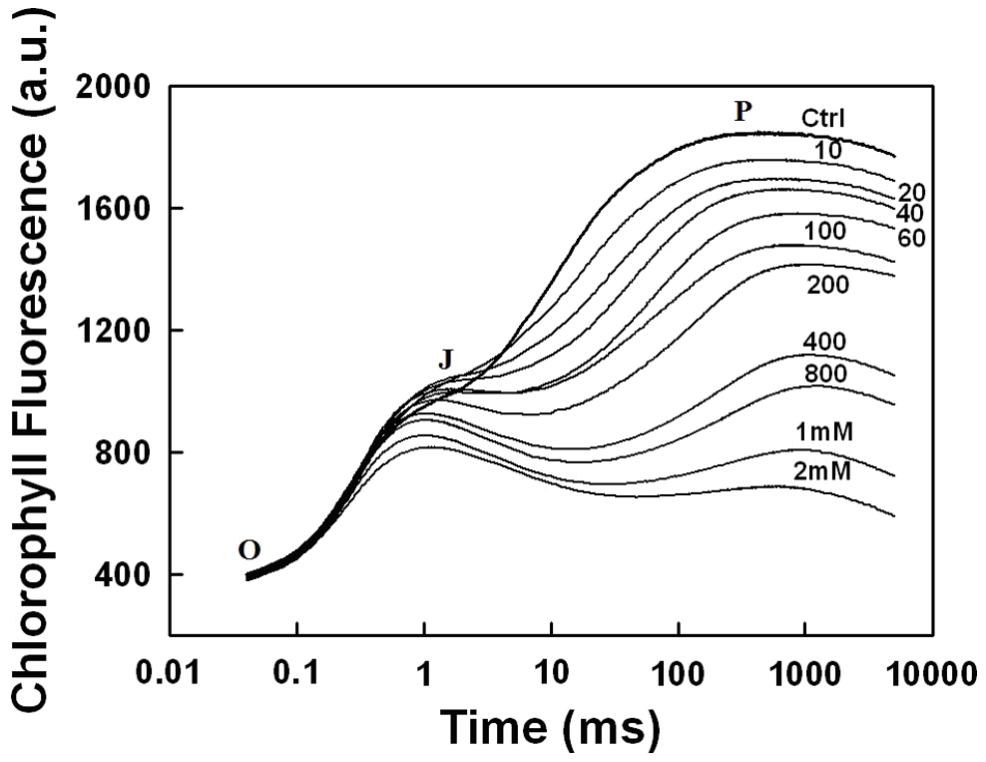
Typical traces of Chl a fluorescence rise in isolated thylakoid membranes in the absence (Ctrl) or in the presence of PbCl_2_ added in various concentrations (µM, unless specified in mM) as indicated by numbers adjacent to traces. See Materials and methods for details.

The kinetic curves of the fast Chl fluorescence rise were measured in isolated thylakoid membranes both untreated and those treated with various concentrations of PbCl_2_ as shown in Fig. 1. The FI traces, normalized at minimal values (F_0_), are characterized by a series of inflections in the rate of rise in the fluorescence intensity termed as OJIP transient (32–33). In isolated thylakoid membranes, the I step of the OJIP fluorescence, as observed by Bukhov et al 2003 (34), cannot be resolved visually due to the significant overlap between JI and IP phases (Fig. 1, Ctrl). However, Pospisil and Dau 2002 (35) and Boisvert et al 2006 (36) have decomposed the OJIP traces by fitting the experimental curves with a sum of three mono-exponential components. This method showed a good fit of the OJIP traces with the three components OJ, JI and IP, even though the inflection step I was absent (36). The I step can be restored by the addition of some different exogenous electron acceptors at the Q_B_ site of PSII (37). The treatment with different concentrations (10–400 µM) of lead increased the relative fluorescence intensity at J step while the rise towards the P step was retarded and the fluorescence intensity at P declined (Fig. 1). This suggests that the electron transfer between Q_A_^−^ and Q_B_ was slowed down with the increasing Pb^2+^ concentration. However, at Pb^2+^ concentrations greater than 400 µM, the OJ phase also began to decrease and the overall fluorescence induction was strongly damped (Fig. 1). Furthermore, the intensity of JIP phase also diminished with increased concentration of Pb cation. In other words, one observed a quenching of Chl fluorescence as the concentration of PbCl_2_ increased.

### Fluorescence of Chl a and MgTPP

**Figure 2:**
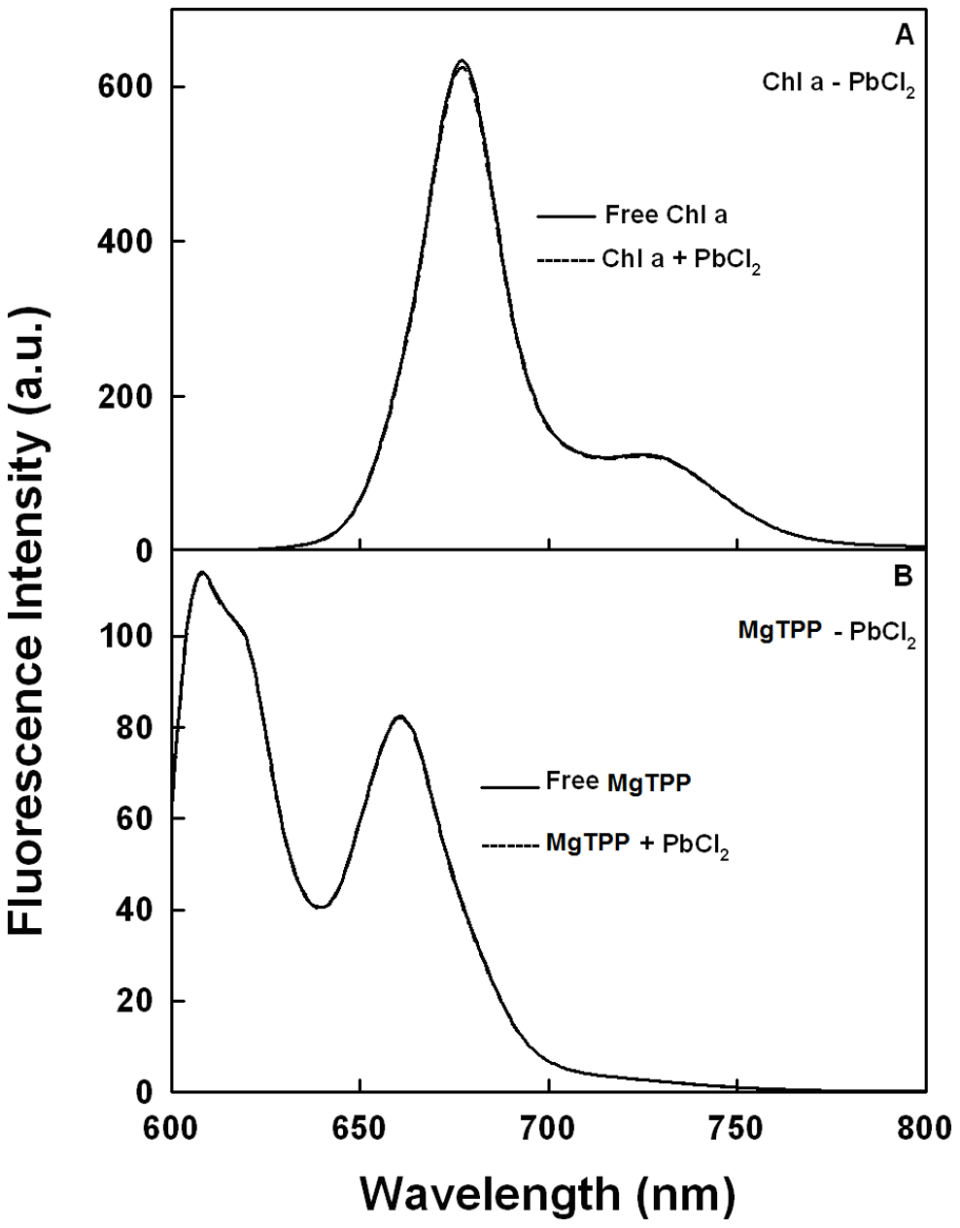
Fluorescence emission spectra of: (A) ethanolic solution of Chl a (2.5 µM) alone (−) and in presence of 1 mM PbCl_2_ (–); (B) ethanolic solution of MgTPP (2.5 µM) alone (−) and in presence of 1 mM PbCl_2_ (–). In both cases, the excitation wavelength was 433 nm.

The observed Chl fluorescence quenching (Fig. 1) may be the result of the direct interaction of PbCl_2_ with the excited states of Chl of PSII thereby altering its radiative characteristics. Therefore, in order to verify this possibility, the fluorescence of Chl a in ethanolic solution was studied in absence and presence of PbCl_2_ (Fig. 2A). The fluorescence of Chl a in this solution exhibits a maximum at 675 nm, which is characteristic of monomeric Chl a (38)**.** As seen from the figure, the fluorescence properties of Chl a practically remained unchanged upon addition of PbCl_2_. This suggests that PbCl_2_ has no direct effect on the excited singlet states of Chl a, and thus on its radiative properties. To further confirm this observation, fluorescence studies of MgTPP were performed in the presence of Pb^2+^ (Fig. 2B). The use of MgTPP is due to its structural analogy with Chl a as it is composed of the same porphyrin macrocycle with central Mg. In addition, the added advantage of using MgTPP is that it does not contain the phytol chain as is present in Chl a, and, as a result, can be more easily accessible to the additives. It is shown in Fig. 2B that PbCl_2_ had no effect on the fluorescence properties of MgTPP thus confirming the results obtained with Chl a.

### Chlorophyll Fluorescence Induction Parameters

**Figure 3:**
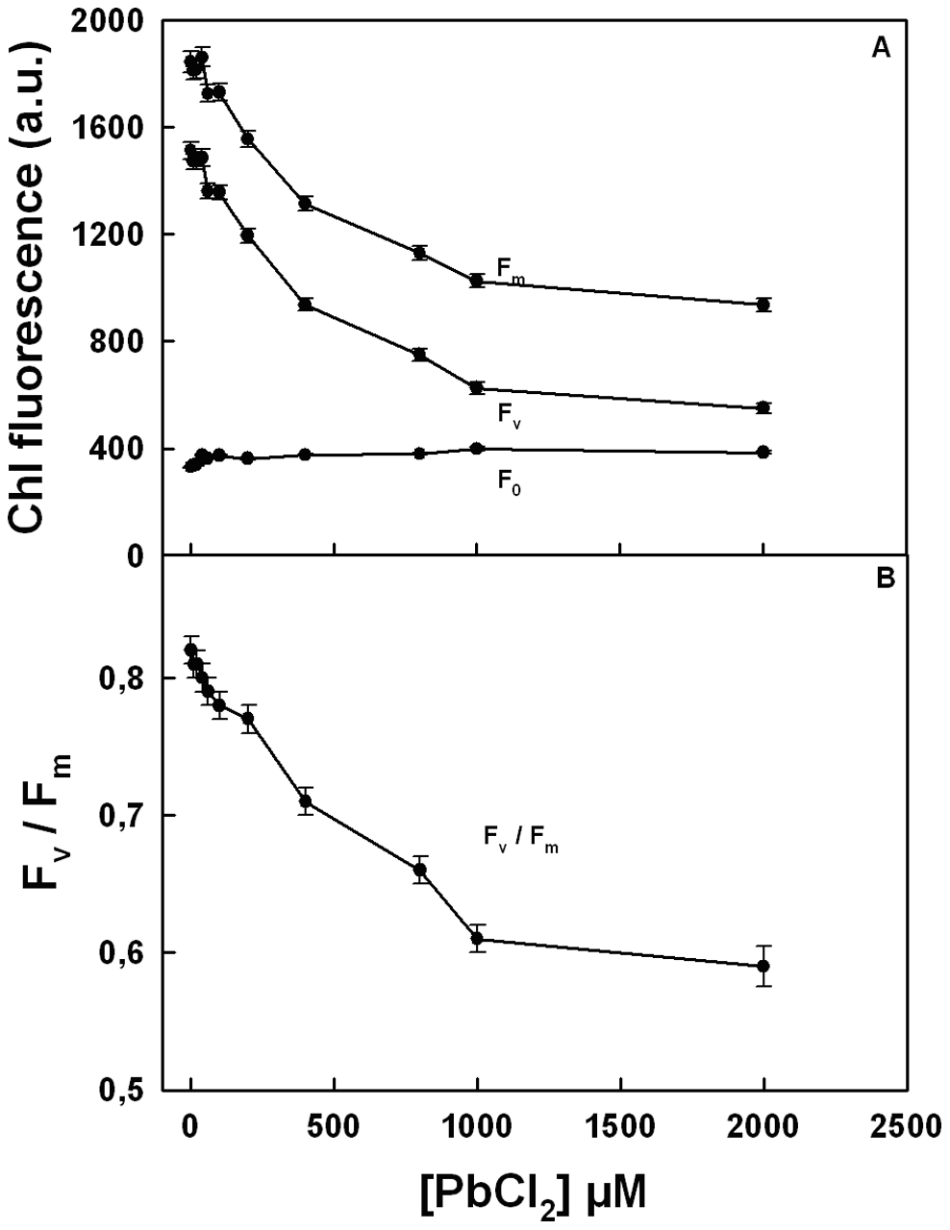
Effect of various concentration of PbCl_2_ on Chl fluorescence induction parameters in thylakoids membranes. (A) F_m_, F_v_ and F_0_, *vs* PbCl_2_ (B) F_v_/F_m_ *vs* PbCl_2_. Each point is the average of nine experiments.

The Chl fluorescence properties of thylakoid membranes in the presence of Pb^2+^ were further subjected to the comprehensive analysis of fluorescence induction kinetics (Fig. 3). The initial fluorescence F_0_ (O-level), which describes the functional state of PSII reaction centers in terms of its openness in the dark-adapted state (39), remained virtually unchanged by the addition of Pb cations (Fig. 3A). However, in order to assess the effect of PbCl_2_ on the maximum quantum yield of the primary photochemistry of PSII in thylakoid membranes, the changes in the maximal fluorescence observed in dark adapted samples, F_m_, when the excitons have been trapped and all the reaction centers of PSII are in closed state (40), were also examined. As seen from Fig. 3A, F_m_ greatly diminished as Pb^2+^ concentration increased. This decline in Fm leads to a decrease in the variable fluorescence Fv (Fv = Fm - F_0_) and, consequently, Fv/Fm, the maximal PSII photochemical quantum yield, also decreased (Fig. 3B). This decrease in Fv/Fm brings about a simultaneous decline in Fv/F_0_ (result not shown), a parameter that accounts for the simultaneous variations in Fm and F_0_ for the determination of the maximum photochemical quantum yield of PSII (41). Since, as noted in Fig. 3A, F_0_ remains practically invariant with increasing Pb concentrations, the inhibitory effect of Pb^2+^ on the quantum yield of PSII photochemistry is, therefore, principally related to the changes in F_m_.

### Flash-induced Chl Fluorescence Decay Kinetics

**Figure 4:**
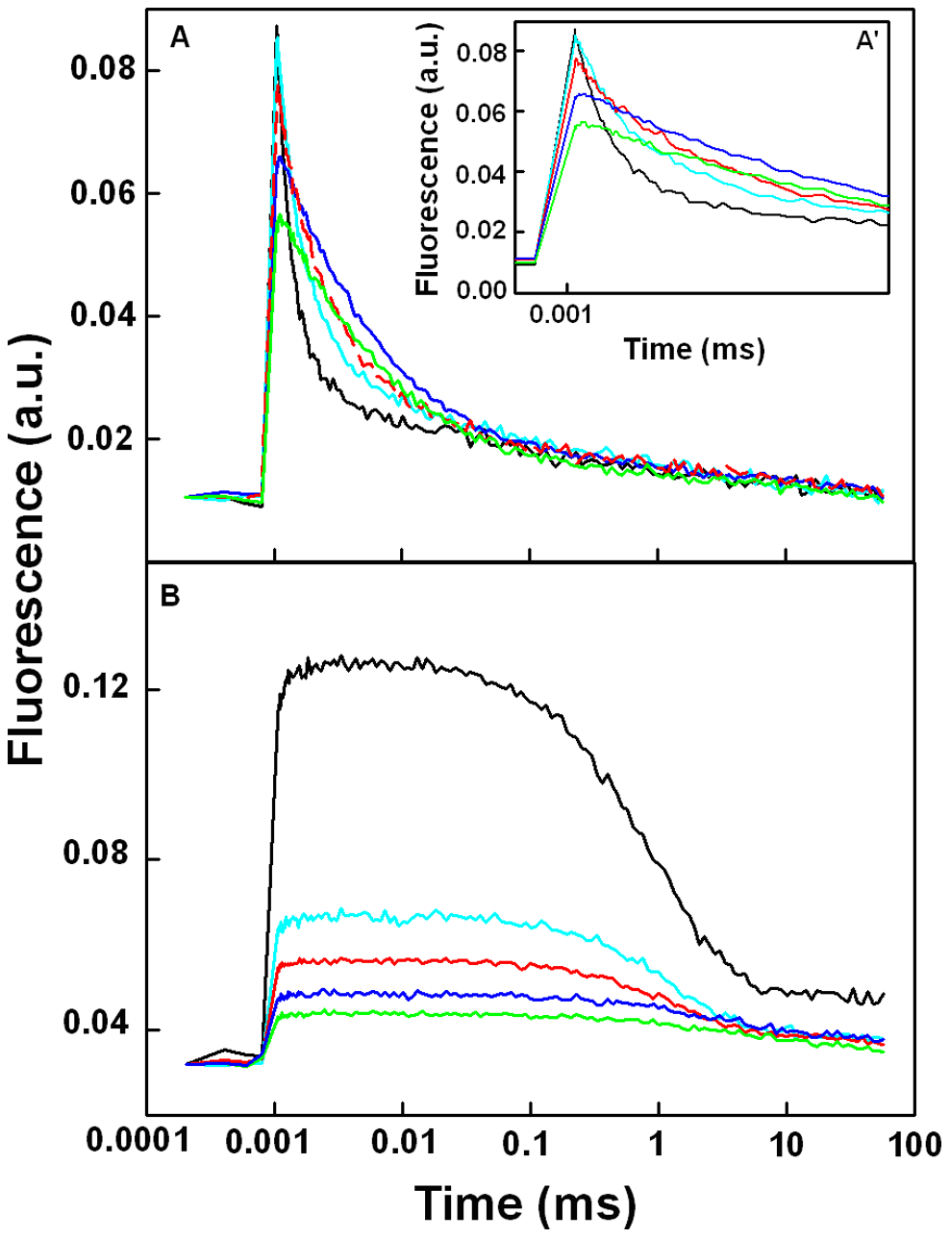
Effect of PbCl_2_ on the relaxation of single turnover flash-induced Chl fluorescence yields in thylakoids membranes with different lead concentrations: 0 (black), 10 (sky blue), 100 (red), 1000 (blue) and 2000 µM (green). The measurements were performed in the absence (A) or in the presence (B) of 50 µM DCMU and all the traces were normalized. Each trace is the average of ten experiments.

The fluorescence induction traces (Fig. 1) indicated that the reoxidation of Q_A_ was affected in the presence of PbCl_2_. This observation was further verified using the fluorescence properties of dark adapted isolated thylakoid membranes submitted to a single turnover flash and normalized at minimal values (Fig. 4A). The fluorescence rise induced by the flash is due to the reduction of Q_A,_ the primary quinone acceptor of PSII, and the decay thereafter in the dark is related to the reoxidation of Q_A_^−^ and consists of several kinetic phases. The amplitude of the fluorescence rise greatly decreased when the concentration of PbCl_2_ increased, especially at concentrations above 100 µM. In order to characterize quantitatively the fluorescence decay kinetics, the dark decay was fitted with three exponential components (Table 1). The fast component is attributed to the reoxidation of Q_A_^−^ by Q_B_ (42–43), the middle component is ascribed to the Q_A_^−^ reoxidation in PSII centers with an empty Q_B_ site and is limited by the diffusion time of PQ to the Q_B_ binding site. The slow phase is associated with the reoxidation of Q_A_^−^ through charge recombination with the S_2_ and/or S_3_ states of the Mn_4_Ca cluster (42–43). The amplitudes and the half-life times of the components are shown in Table 1. The amplitude of the fast phase greatly decreased with increasing amounts of PbCl_2_, this was accompanied by a strong increase in the half-life time of all three components of the decay kinetics (Table 1).

**Table 1:** Effect of PbCl_2_ on the relative amplitude (A) and half-life time (t_1/2_) of the exponential decay components of Chl a fluorescence yield after a single turnover flash measured in thylakoid membranes in the absence and in the presence of 50 µM DCMU.

Without DCMUPbCl_2_ (µM)	Fast Phase		Middle Phase		Slow Phase	
	t_1/2_ (±10 µs)	A (±5%)	t_1/2_ (±0.3 ms)	A (±3%)	t_1/2_ (0.4 s)	A (±2%)
0	430	69	6.1	22	5.9	9
10	690	61	7.5	26	7.1	13
100	987	45	11.3	35	12.1	20
1000	1440	28	16.8	43	15.6	29
2000	1590	19	18.2	46	17.2	35
**With DCMU** **PbCl_2_ (µM)**	**Fast Phase**		**Middle Phase**		**Slow Phase**	
	**t_1/2_ (±5** **ms)**	**A (±4%)**	**t_1/2_ (±0.03** **s)**	**A (±3%)**	**t_1/2_ (±0.3** **s)**	**A (±2%)**
0	298	41	1.79	47	18.5	12
10	1130	26	1.92	53	22.1	21
100	1560	19	2.25	58	23.6	23
1000	–	–	4.35	68	31.2	32
2000	–	–	4.86	69	31.9	31

The decay was also measured in the presence of DCMU that blocks electron transfer between Q_A_^−^ and Q_B_ (Fig. 4B). In this case, the reoxidation of Q_A_^−^ is owing to its charge recombination with the donor side of PSII. The fluorescence decay in the presence of DCMU can also be fitted with three exponential components (44–45). The fast component is due to the charge recombination with partially active Mn_4_Ca clusters, the middle component arise from the recombination of S_2_Q_B_^−^ charge pairs. The slowest component is associated with PSII with an oxygen evolving center in the S_0_ state before the flash was applied. The amplitudes of the middle and slow components increased with PbCl_2_ at the expense of the fast component and their half-life times increased (Table 1).

### Thermoluminescence

**Figure 5:**
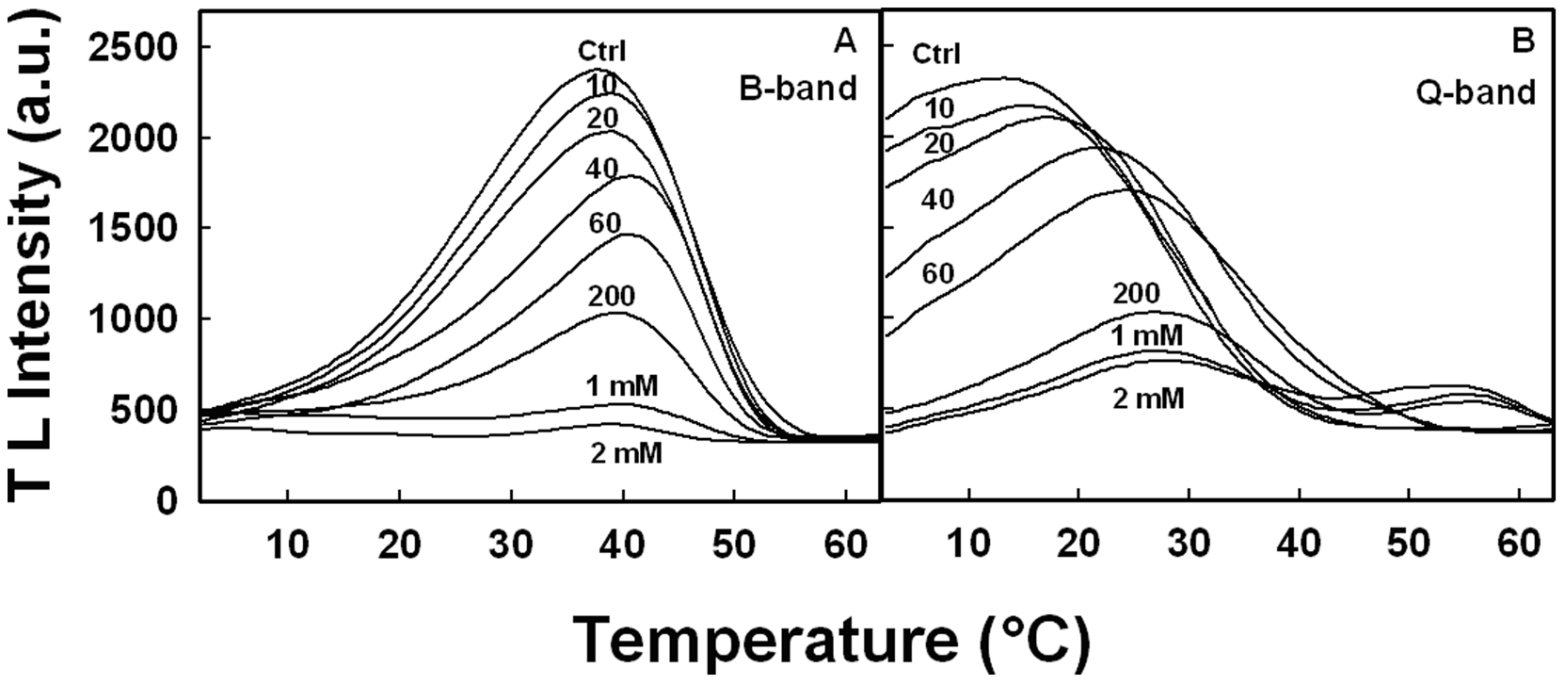
Thermoluminescence spectra measured in isolated thylakoid membranes in the absence (A) or presence of 50 µM DCMU. PbCl_2_ was added in various concentrations (µM, unless specified in mM) as indicated by numbers adjacent to traces.

Thermoluminescence was used to further explore the effects of PbCl_2_ on charge recombination between donor and acceptor sides of PSII. The TL glow curves for untreated (Ctrl) and Pb^2+^-treated thylakoid membranes following two single turn-over white flashes are displayed in Fig. 5A. The TL signal (Fig. 5A, Ctrl) attained its maximal intensity at the temperature of 38°C, characteristic of the temperature optimum for the B band appearing in the range between 30 and 40°C, as previously reported for this type of material (46–47). The B band is attributed to the charge recombination of S_2_/S_3_Q_B_^−^ pairs produced by linear electron transport in PSII (48–51). The intensity of the B band progressively diminished as the concentration of PbCl_2_ increased (Fig. 5A). The addition of 20 µM PbCl_2_ produced 13% decrease in TL intensity, and in the presence of 2 mM PbCl_2_, the TL intensity was suppressed completely. Also, the decline of the band was accompanied by an upshift of the maximal temperature (Tm) from 38°C to 41°C.

The changes in the amplitude and Tm of the B band could be related to changes in the properties of the S_n_ states of the Mn_4_Ca cluster and/or to modification of the Q_B_ binding site in the presence of PbCl_2_. In order to elucidate the site of action of PbCl_2_ in the electron transport chain, the TL glow curves were recorded following two single turn-over white flashes in the presence of 50 µM of PSII inhibitor DCMU, known to block the electron flow beyond Q_A_. DCMU eliminated the B band with a simultaneous appearance of Q band with a maximum at 17°C, attributed to the back-flow of electrons from Q_A_^−^ to the S_2_-state (48–51) (Fig. 5B). The reason for the absence of B band is that since DCMU stops the electron flow past Q_A_, the formation of Q_B_^−^ and state S_3_ is not realized (49). The addition of 20 µM PbCl_2_ already suppressed 12% of the Q band intensity. A progressive decrease of the Q band was observed when the concentration of PbCl_2_ was further increased. The addition of 2 mM PbCl_2_ caused the loss of more than 90% of Q band intensity. This loss was accompanied by a strong upshift of Tm from 17°C to 27°C.

## Discussion

The negative action of Pb^2+^ on PSII photochemistry and electron transport, uninfluenced by PSI activity, was studied in thylakoid membranes using various approaches specific for PSII. Chlorophyll fluorescence induction kinetics measurements (Fig. 1) have shown that the fluorescence was greatly quenched when PbCl_2_ was added. Several authors postulated that damage caused by heavy metal ions (such as Zn^2+^, Cu^2+^, and Pb^2+^) to plants was due to the substitution of the central Mg from the Chl a molecules thus causing fluorescence quenching (52–54, 22). However, measurements of pure Chl a or MgTPP fluorescence in ethanolic solution (Fig. 2) have demonstrated that the addition of PbCl_2_ has no effect on the excited states of Chl a and the structure of the pigment remains intact. The fluorescence quenching observed during Chl fluorescence induction is, therefore, related to the modifications in the photochemical activity of PSII.

The OJIP traces constitute an essential tool to study the activity and integrity of the photosynthetic apparatus under different stress conditions, providing the information on PSII photochemistry such as the electron transport on both donor and acceptor sides of the photosystem (32–33, 55). The IP step of the Chl fluorescence induction has been correlated with the photoreduction of the PQ pool (56–57). Thus, the observed decline in IP phase indicates a strong inhibition of the accumulation of reduced PQ especially at Pb^2+^ concentrations above 400 µM (Fig. 1). This coincided with a decrease in the Fv/Fm values due to a decrease in Fm (Fig. 3) (23). This part of the induction is known to be more sensitive to the unfavourable treatments in comparison with the photochemical phase (OJI) (58). Indeed, the perturbation in the structure-function relations of the WOC has been shown to correlate with the quenching of the IP fluorescence rise that results in a decline of Fm (36, 58). The above is in line with the previous reports showing that Pb^2+^ causes the release of extrinsic polypeptides associated with the WOC together with the Ca^2+^ and Cl^−^ required as cofactors (5, 25). Therefore, the inhibition of JIP rise and the more significant damping of the whole fluorescence induction kinetics above 400 µM Pb^2+^ are the result of the disorganization of the WOC causing the lack of electron flow towards the acceptor side of PSII. The damage of the Mn_4_Ca cluster is also supported by the decline of both Q and B thermoluminescence bands. Such inhibition of both S_2_Q_A_^−^ and S_2_Q_B_^−^ charge recombination (Q and B band, respectively) shows that the S_2_ state of the WOC becomes unavailable as the common recombination partner with increasing concentrations of PbCl_2_ and indicates a dysfunction of the WOC.

On the other hand, the OJ phase is related to the reduction state of Q_A_ (56, 59). The relative increase of OJ in the presence of low concentrations of PbCl_2_ (Fig. 1) is strongly indicative of a delayed electron transfer from Q_A_^−^ to Q_B_. This was indeed verified using the measurements of Chl fluorescence decay kinetics following a single turn-over flash (Fig. 4). The fluorescence decay was greatly retarded with the life-time of all three components being significantly increased even at concentrations below 400 µM PbCl_2_ (Table 1). The amplitude of the fast component, attributed to electron transfer from Q_A_^−^ to Q_B_, diminished with a concurrent increase of the other components. Also, the decreased rate of Q_A_^−^ reoxidation resulted in an increased amplitude of the slow component attributed to the back reactions with the S_2_ state of the Mn_4_Ca cluster (42–43). This corresponds with the increased amplitude of the middle component of the decay measured in the presence of DCMU (Table 1), a component also attributed to S_2_/Q_A_^−^ recombination (44–45). Therefore, the population of PSII centers with a reduced Q_A_ that is reoxidized through S_2_/Q_A_^−^ recombination is increased but the rate of this reoxidation is strongly declined most likely due to the stabilization of the S_2_ state of the WOC (see below).

**Figure 6:**
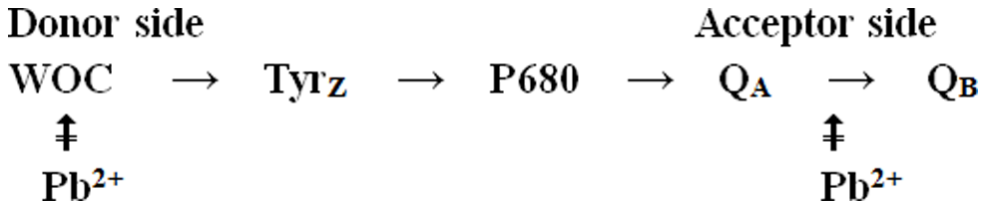
Schematic representation of the proposed inhibitory sites of Pb^2+^ in PSII.

The delayed reoxidation of Q_A_^−^ maybe interpreted in terms of an active site of Pb^2+^ being near Q_A_ or Q_B_. Indeed, similar data were previously used to conclude that an inhibitory site of various metal cations was located between Q_A_ and Q_B_ (Fig. 6) (60–62). However, the destabilization of the WOC discussed above may also cause the delayed Q_A_^−^ reoxidation. It was indeed shown that the removal of the extrinsic polypeptides or Ca^2+^ from the WOC can cause the diminished rate of Q_A_^−^ reoxidation through a transmembrane conformational effect (42). Removal of Ca^2+^ from the WOC also produces a modification in the mid-point potential of Q_A_ thus altering the electron transfer process between Q_A_ and Q_B_ (63, 43). It can be postulated that this conformational change modifies the bicarbonate binding that is required for proper electron transfer from Q_A_^−^ to Q_B_ (17, 18). Therefore, it is plausible that the action of Pb^2+^ at the WOC would cause this same transmembrane effect as was also proposed for the inhibitory action of Ni^2+^ and polyamines (64–65). This view is supported by the strong progressive upshift of the Tm of Q and B thermoluminescence bands with increasing concentrations of Pb^2+^ (Fig. 5). Such large increase in thermoluminescence temperature was previously associated with the stabilization of the S_2_ state of the WOC due to the modification in the ligand environment of the Mn_4_Ca complex following the depletion in Cl^−^ or in 33 kDa extrinsic polypeptide (66–67). Therefore, the shift of Tm towards higher temperatures may be due to a change in the population of PSII centers with a stabilized S_2_ state owing to the action of Pb^2+^ causing a retarded Q_A_^−^ reoxidation at low Pb^2+^ concentrations. This may represent an intermediate step in the inhibition of the WOC that precedes the serious damping of the fluorescence induction observed at high Pb^2+^ concentrations (Fig. 1).

Although an active site of Pb^2+^ at or near Q_B_ cannot be fully excluded, the negative action of Pb^2+^ is postulated to proceed in two steps. During the intermediate step, the environment of the Mn_4_Ca complex is disorganized and the S_2_ state of the WOC is stabilized which consequently affects Q_A_^−^ reoxidation and increases S_2_/Q_A_^−^ charge recombination (though the recombination proceeds at a slower rate compared to the control). During the final phase, the WOC is damaged more seriously leading to a loss of charge recombination and of PQ reduction.
